# The pioneering work of Howard Cedar and his team in unraveling the roles DNA methylation plays in regulating genome function

**DOI:** 10.3389/freae.2026.1754553

**Published:** 2026-04-14

**Authors:** Eugin Destici, Eric Mendenhall, Alon Goren

**Affiliations:** 1 Department of Medicine, University of California, San Diego, CA, United States; 2 HudsonAlpha Institute for Biotechnology, Huntsville, AL, United States

**Keywords:** chromatin, development, DNA methylation, gene regulation, genome

## Abstract

The covalent modification of specific DNA nucleotides by addition of a methyl group, termed DNA methylation, was observed in bacteria in 1925 and in mammals in 1948. Yet, the function of this modification was uncertain for many years, particularly its roles in mammalian cells. During the past 5 decades, work from Howard Cedar and his lab has provided fundamental and groundbreaking insight into the role DNA methylation plays in regulating gene expression in health and disease. Using simple yet elegant experiments, Cedar and his team provided unequivocal evidence for the direct role of DNA methylation in transcription regulation, and demonstrated that DNA methylation serves as a stable heritable epigenetic mark. Building on these foundational discoveries, Howard Cedar and his team have further illuminated the critical role of DNA methylation in organismal development and cellular function, as well as the contribution of altered DNA methylation to cancer. Further, Cedar and his lab also demonstrated that the developmentally regulated removal of DNA methylation is pre-programmed in the genome via sequence motifs that recruit trans-acting factors at specific developmental phases. Howard Cedar’s pioneering scientific contributions have resulted in paradigm shifts in the study of transcription as well as DNA replication and set up the groundwork for fields of research that continue to thrive and expand into areas such as diagnostics, modeling of aging and targeted treatments.

## Introduction

Chemical modifications to the DNA and histones in eukaryotes are now known to be key to genome packaging and the regulation of gene expression, influencing virtually every cellular process. These modifications can affect transcription through a variety of mechanisms, leading to positive and negative transcriptional effects depending on the type of modification and context.

Following the initial observations of the presence of DNA methylation in bacteria (Johnson and Coghill, 1925) and later in mammalian cells (Hotchkiss, 1948), it was discovered that in bacteria, DNA methylation protected the genome from host restriction enzymes. Yet, for a long time the prevailing hypothesis was that DNA methylation played no major role in mammalian cells (Mattei et al., 2022). Early studies by Riggs and Holliday and Pugh put forth the hypothesis that DNA methylation was associated with repression of gene expression but evidence for a direct effect was lacking (Holliday and Pugh, 1975; Razin and Cedar, 1984; Riggs, 1975). In particular, studies on β-globin gene expression, which is specifically expressed in blood cells, indicated that the locus was unmethylated in red blood cells but extensively methylated in tissues that lack β-globin gene expression (Cedar et al., 1983; McGhee and Ginder, 1979).

More generally, DNA methylation is broadly associated with stabilizing the repression of gene expression and is established by the *de novo* DNA methyltransferases DNMT3A and DNMT3B (Okano et al., 1999). Methylation can be erased passively through replication, or through an active mechanism involving the TET enzymes (Tahiliani et al., 2009). DNA replication produces hemi-methylated DNA molecules (Figure 1) which are recognized by UHRF1 (Bostick et al., 2007; Sharif et al., 2007) and then becomes fully methylated by DNMT1 (Bestor et al., 1988). Recent work has also hinted at a role for DNMT1 in *de novo* DNA methylation (Haggerty et al., 2021), the authors noted that the DNMT1/UHRF1-dependent *de novo* methylation occurred in particular at specific retrotransposon elements (Haggerty et al., 2021) that need to remain silenced to ensure genome integrity. These elements are often targets of DNA methylation (Bestor and Bourc’his, 2004; Deniz et al., 2019). DNA methylation can both repel and attract binding of regulatory proteins (Hu et al., 2013; Spruijt et al., 2013; Yin et al., 2017). A classic example of a protein that specifically binds methylated DNA is MeCP2, which is mutated in Rett syndrome (Good et al., 2021; Kriaucionis and Bird, 2003). Another layer of epigenetic control is provided by histone modifications, which in some cases are intimately linked to DNA methylation (Allis and Jenuwein, 2016; Cedar and Bergman, 2009). Specific examples of this include the recruitment of DNMT3A/B by the methylation of lysine 36 on H3 (H3K36me2/3) at genic (Baubec et al., 2015; Morselli et al., 2015) as well as intergenic regions (Weinberg et al., 2019) leading to DNA methylation of regions marked by that histone modification as well as repulsion of the DNA methylation machinery by the promoter mark H3K4me3 (Morselli et al., 2015; Ooi et al., 2007). Furthermore, direct binding of the heterochromatin mark H3K9me2/3 by UHRF1 contributes to DNMT1-mediated DNA methylation and epigenetic repression (Liu et al., 2013; Rothbart et al., 2012).

**FIGURE 1:**
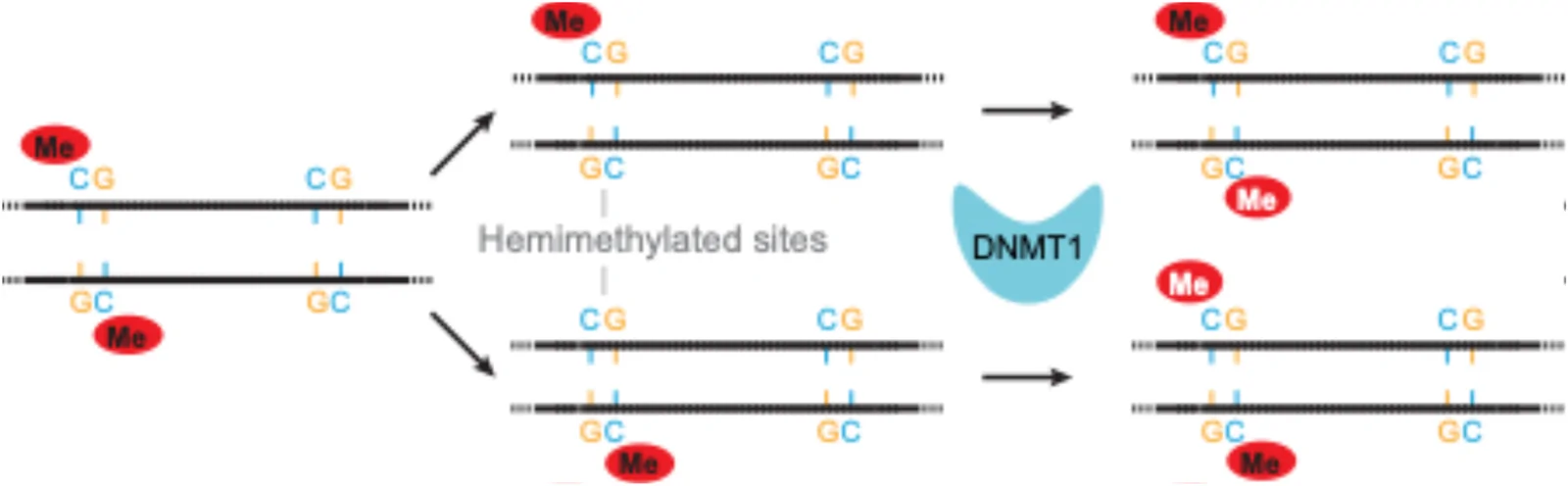
A model depicting the mechanism for maintenance of DNA methylation following DNA replication. Each replicated region inherits one methylated daughter strand leaving the DNA hemimethylated. In the absence of further DNA methylation this would result in the erasure of DNA methylation after successive rounds of replication. To prevent this erasure, the maintenance DNA methylase DNMT1, as part of a larger complex (see text), ensures faithful maintenance of DNA methylation by methylating the other unmethylated strand.

While many other labs have made significant contributions to the elucidation of the role of DNA methylation, this Perspective will focus on the work of just one of the trailblazers in this field, Howard Cedar and his group, and refer the reader to other excellent reviews that provide more comprehensive and historical insight into the field (Bestor et al., 2015; Bird, 2002; Greenberg & Bourc’his, 2019; Holliday, 2006; Luo et al., 2018; Mattei et al., 2022; Stelzer and Jaenisch, 2015). As described below, by conducting fundamental work studying the role DNA methylation plays in regulating and stabilizing gene expression in health and disease, Howard Cedar and his team provided the foundation for the current understanding of this crucial regulatory mechanism.

## Cedar’s interdisciplinary background

Howard Cedar obtained a BSc degree in mathematics from MIT and then transitioned to an MD-PhD at NYU. As discussed below, Cedar has built on his (quite unusual in those days) interdisciplinary background, and in recent years many of his group’s work has included genomic analysis (e.g., Falick Michaeli et al., 2024; Falick Michaeli et al., 2022). Following his graduation from NYU, Cedar joined the Lab of Gary Felsenfeld at NIH (Cedar, 2014), a pioneer in the field of gene regulation and epigenetics. At the Felsenfeld Lab, Cedar was introduced to the emerging field of genetics and the biology of chromatin–the complex of DNA and associated proteins. While many labs at the time used naked DNA templates to study transcription, Felsenfeld had developed a technique to solubilize chromatin (Cedar and Felsenfeld, 1973) facilitating the use of chromatin templates (as opposed to naked DNA) to study transcription and the role of chromatin in this process (Cedar, 2014; Cedar and Felsenfeld, 1973).

At the Felsenfeld lab, Cedar worked alongside Richard Axel, who went on to win a Nobel Prize for his work identifying olfactory receptor genes. Together, they used solubilized chromatin to study its impact on transcription *in vitro*. In the early days of gene regulation studies, the role of chromatin (which was initially discovered in the 19th century) was overlooked and chromatin was mainly considered as a means of compacting DNA to fit inside the nucleus. The existence of chromatin modifications was established in the 60s (Allfrey et al., 1964) but a clear connection to transcription was still lacking. Biochemical studies from Axel and Cedar while in the Felsenfeld lab indicated that chromatin prevents pervasive transcription of DNA, suggesting that chromatin adds an additional layer of transcriptional control that can be utilized for tissue-specific gene expression (Axel et al., 1973). These pioneering studies, along with contemporaneous work from other labs (O'Malley et al., 1977; Parker et al., 1978), demonstrated that chromatin plays a key role in regulation of gene expression.

## Demonstration of a direct effect of DNA methylation on gene expression

After moving to Israel in 1973, Cedar teamed with Aharon Razin who was working down the hall, and they developed a long-lasting collaboration (Cedar, 2014). Razin was studying bacterial DNA methylation systems where each bacterial strain has its own methylation system that targets different DNA sequences for methylation. DNA methylation protects these sites from cleavage by the host bacteria’s restriction enzyme. However, when bacteria encounter foreign DNA from other bacteria or phages, these regions are unmethylated, allowing the invader’s DNA to be cut by the host restriction enzyme (Razin and Friedman, 1981). In contrast, it was not clear what the role of DNA methylation was in mammalian cells. Cedar and Razin, alongside others (Holliday and Pugh, 1975; Riggs, 1975), hypothesized early on that DNA methylation in mammals might play a role in gene regulation (Cedar, 2014).

In 1977, Cedar went to the lab of Richard Axel for a sabbatical and learned their new DNA transfection methods (Wigler et al., 1977). Back in Israel, Cedar and Razin designed a simple yet elegant and conclusive experiment–a DNA construct was methylated *in vitro* using bacterial DNA methyltransferases, then introduced into mammalian cells using DNA transfection to assess expression of a reporter gene. These experiments were performed prior to the usage of selection markers such as neomycin or puromycin. Thus, to ensure chromosomal integration of the reporter plasmids, the team used a mouse fibroblast cell line lacking both tk^-^ and aprt^-^ and conducted co-transfection of plasmids encoding TK (Wigler et al., 1977) and the hamster Aprt gene (Stein et al., 1982b). By using TK gene integration as a selectable marker for cells that integrated plasmids, they were able to use azaserine-containing media to assess Aprt expression (Figure 2).

**FIGURE 2:**
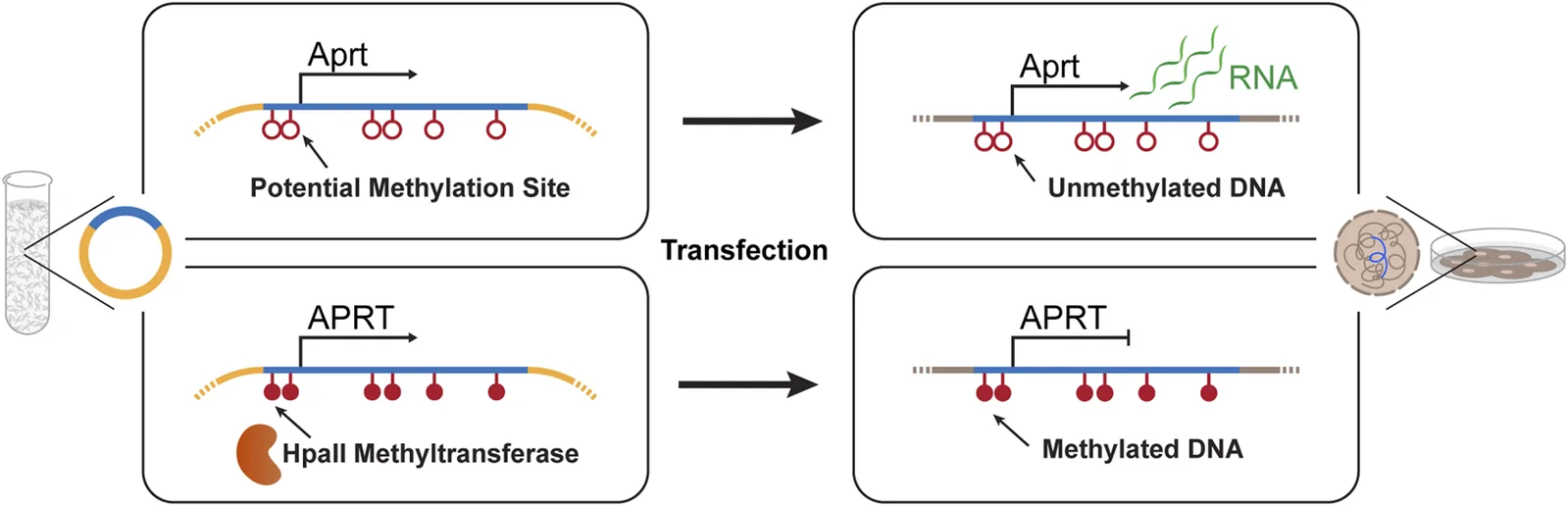
A schematic illustrating the key experiment showing a direct effect of DNA methylation on gene expression (Stein et al., 1982b). A reporter plasmid is methylated *in vitro* or left unmethylated and then transfected together with a TK resistance plasmid (not shown for simplicity) facilitating chromosomal integration of the plasmids, based on methods developed by the lab of Richard Axel. Stein et al. demonstrated that both methylated and unmethylated plasmids were efficiently transfected and integrated into the genome but only the unmethylated plasmid was able to drive robust expression demonstrating a direct (repressive) effect of DNA methylation on gene expression.

The key part of this experiment was that the Aprt plasmid was either methylated *in vitro* or unmethylated before transfection into the cells. While methylated and unmethylated plasmids were both efficiently integrated into the genome, the methylated plasmid was transcriptionally silent, shown by cell death in azaserine media, while Aprt was robustly expressed from the unmethylated plasmid (Stein et al., 1982b) providing strong evidence for a direct cis-regulatory effect of DNA methylation on gene expression. Additionally, Aprt expression stayed off for over 50 cell divisions, further supporting that DNA methylation can be stably inherited during mammalian DNA replication.

This observation was confirmed in another study from Cedar’s lab at around the same time, using replication-competent viral DNA transfected into mouse cells (Stein et al., 1982a). These results suggested a way for gene expression control to be transmitted across cell divisions, a true example of epigenetic regulation. Over the years, Cedar and his colleagues have further explored this concept in more physiological contexts. These include the study of the developmental silencing of the master pluripotency regulator Oct4, which is initiated by trans acting factors but only becomes stable after the recruitment of the *de novo* DNA methylation machinery (Epsztejn-Litman et al., 2008; Feldman et al., 2006). In other studies Cedar and his team showed how environmental impact brings about permanent changes to DNA methylation and gene expression. For instance they demonstrated how specific hormones or factors lead to changes that are stabilized via DNA methylation during liver regeneration (Reizel et al., 2015) and in response to muscle injury (Falick Michaeli et al., 2022).

## Defining the mechanistic roles DNA methylation plays in modulating chromatin structure in development and disease

Building on these foundational findings and techniques, Cedar and colleagues made several additional pioneering discoveries. Using DNase hypersensitivity assays combined with the *in vitro* methylation and the transfection techniques mentioned above (Cedar et al., 1983) they demonstrated that DNA methylation decreased chromatin accessibility (Keshet et al., 1986). They built on these results and extended the observations using MNase-based assays to show that DNA methylation caused the chromatin to be in an inactive state (Keshet et al., 1986). Notably, these findings still held up in genome level studies (Battaglia et al., 2022; Lee et al., 2020; Meissner et al., 2008; Ziller et al., 2013).

To interrogate the involvement of DNA methylation in cell-type-specific gene expression regulation, Cedar and his team transfected methylated or unmethylated constructs into different mammalian cell types. They used the promoter region of the α-actin gene, which was shown to be sufficient to drive muscle-specific reporter activity (Melloul et al., 1984). Similar to their previous results, the methylated plasmid was inactive when transfected into fibroblasts. However, in myogenic cells, which normally express α-actin, they observed that the *in vitro* methylated reporter plasmid became active. Further experiments uncovered that the methylated reporter plasmid underwent site-specific demethylation in the myogenic cells providing the first example of a site- and cell-type-specific demethylation system (Yisraeli et al., 1988). This experimental validation of *in vivo* demethylation events further corroborated that cell type specific genes can undergo site-specific DNA demethylation in the cell type in which they are expressed (Yisraeli et al., 1988).

Follow-up work interrogated the dynamics of this demethylation process, demonstrating that DNA demethylation preceded cell-type-specific gene activation (Paroush et al., 1990). This study also demonstrated that specific DNA-elements were required for site-specific DNA demethylation and provided evidence for the replication-independent nature of this process (Paroush et al., 1990). While DNA was known to undergo passive DNA demethylation in a replication-dependent manner (Kohli and Zhang, 2013), these striking observations, together with work from others, opened the field to a decades-long quest to find the mammalian DNA demethylase culminating in the discovery of the TET enzymes in 2009 by the lab of Anjana Rao (Tahiliani et al., 2009).

Later work from Cedar’s lab focused on studying how tissue-specific or stage-specific demethylation occurs during development. These studies demonstrated the involvement of tissue- or stage-specific trans-acting factors that bind to specific motifs in cis-regulatory regions. These tissue/stage-specific trans-acting factors then recruit the DNA demethylation machinery. Once DNA demethylation at these specific regions is established, this, like DNA methylation, is stably maintained even in the absence of the trans-acting factors (Orlanski et al., 2016; Reizel et al., 2018; Reizel et al., 2015).

Cedar continues to work on DNA methylation throughout his long and fruitful career, and to make groundbreaking discoveries using new technologies. Using a clever approach to generate a reporter construct whose methylation pattern can be controlled in mouse models allowed Cedar and his team to study the effect of site-specific DNA methylation during normal development. They first demonstrated that an embryo-specific DNA sequence-element upstream of the Aprt gene prevents DNA methylation of itself and immediately adjacent sequences (Siegfried et al., 1999). Next, they went on to use this sequence-element to generate a removable construct, thus allowing control of the methylation state of a transgene reporter in mice. Using this elegant system, the Cedar lab established that DNA methylation repressed gene expression in cis in different tissues *in vivo* (Siegfried et al., 1999), building and expanding on their previous studies that utilized cell lines. This system was also used to dissect the role of DNA methylation in the cell-type specific developmental regulation of the human β-globin gene cluster which encompasses both fetal and adult genes by using a transgenic mouse model. Using this approach, Cedar and his team showed that DNA methylation serves as a means for selecting the appropriate globin genes during development (Goren et al., 2008).

Together with Yehudit Bergman, another investigator with whom Cedar has a decades-long and very fruitful collaboration, Cedar interrogated the involvement of DNA methylation in immunoglobulin gene rearrangement and allelic exclusion (Farago et al., 2012; Ji et al., 2003; Lichtenstein et al., 1994; Mostoslavsky et al., 1998) providing not only novel insight into this key immune cell process but also demonstrating a role for DNA methylation beyond transcription.

Shifting to cancer, the Cedar lab developed a new microarray-based technique to map DNA methylation genome-wide (Keshet et al., 2006). Using this assay, they set out to map the DNA methylation landscape to interrogate whether tumor suppressor genes undergo random silencing, which is then selected for during tumorigenesis, or whether an instructive pathway exists that targets specific genes or genomic regions for silencing. Their genome-wide datasets revealed that methylated genes in cancer cells were not randomly located throughout the genome but rather tended to cluster and were associated with specific functional categories, illuminating an instructive mechanism driving *de novo* methylation in cancer (Keshet et al., 2006).

While the broad body of work discussed above clearly demonstrates a key role for DNA methylation as an epigenetic regulator of gene expression in a variety of contexts, it does not address how a cell decides which specific cytosines get methylated. This question was also investigated by Cedar and his team, and they were able to demonstrate that DNA methylation patterns are encoded in the DNA through regulatory motifs that are bound by cell-specific and state-specific trans-acting factors. The activity of these cell/state specific trans-acting factors then leads to DNA methylation patterns that reflect the unique gene expression profiles of the different cells (Greenfield et al., 2018; Sabag et al., 2014; Straussman et al., 2009).

In addition to his work on the role of DNA methylation in gene expression, Cedar’s work also provided extensive insight into DNA replication timing and the epigenetic mechanisms involved. In one early example, Cedar and colleagues helped illuminate the involvement of DNA methylation in the control of replication timing (Selig et al., 1988). Not long after, Cedar’s lab developed a method to identify mammalian origins of replication (ORIs) and utilized this method to identify and validate several ORIs (Handeli et al., 1989). They also demonstrated that imprinted genes display allele-specific replication timing (Kitsberg et al., 1993) and revealed that this is established soon after fertilization and is maintained throughout development (Simon et al., 1999). Cedar’s lab further investigated the epigenetic mechanisms involved in replication timing. In one example, artificial tethering or histone acetyltransferases of deacetylases utilizing mouse transgenic models demonstrated that histone acetylation of ORIs serves as a switch controlling replication timing *in vivo* (Goren et al., 2008). In other work, the Cedar lab used microinjection of exogenous DNA into nuclei and demonstrated that DNA inserted into early S-phase nuclei displayed higher levels of transcription when compared to the same DNA injected into late S-phase nuclei. Further analysis showed that the differential transcriptional competence observed was associated with distinct chromatin states of the injected DNA, thus demonstrating a causal and mechanistic link between replication timing and transcription (Zhang et al., 2002).

Consistent with the critical role DNA methylation plays in gene expression control, Cedar’s team elucidated the key role of DNA methylation in reprogramming of cells, both in context of early development in the embryo (Naama et al., 2023) as well as in the context of trans-differentiation through expression of exogenous TFs (Radwan et al., 2024), ensuring the stability of reprogramming through stable control of gene expression. Furthermore, their work provided insight into how aberrant DNA methylation contributes to cancer and aging in humans (Klutstein et al., 2017; Klutstein et al., 2016).

## Broader current understanding of the implications of DNA methylation

The understanding of DNA methylation has come a long way from its initial discovery as a biochemical curiosity a century ago, propelled by the work of Cedar and many other labs that followed in his footsteps. For instance, work from Cedar and others has shown that DNA methylation can be used diagnostically for the early detection of solid cancers and cancer progression as well as for some other diseases using blood samples. Using cell-free circulating DNA blood tests allows detection of specific DNA methylation patterns that can be used to indicate the presence of cell death and the tissue from which it has emerged (Dor and Cedar, 2018; Lehmann-Werman et al., 2016). Moreover, demethylating agents such as 5-azacytidine, which was initially discovered as a differentiation agent (Jones and Taylor, 1980) before it was known to inhibit DNMTs, have been used clinically for decades to treat certain types of cancer (Rivard et al., 1981; van Groeningen et al., 1986). DNA methylation has also been used as an epigenetic clock to measure aging across tissues in humans (Horvath, 2013). Remarkably, recent work on the β-globin locus supports the notion that reversal of DNA methylation of specific sites in the β-globin locus may be used therapeutically for hemoglobinopathies (in which adult globin genes are mutated) by increasing expression of fetal globin at the expense of adult globin (Bell et al., 2025). In other recent work to interrogate human hematopoiesis, stable patterns of DNA methylation were used for clonal cell tracing *in vivo* (Scherer et al., 2025), which normally requires transgenes or genome engineering tools that are only applicable to model organism or *in vitro* experiments. Lastly another recent study used DNA methylation barcodes that fluctuate over time to study the dynamics of cancer development (Gabbutt et al., 2025).

## Lasting legacy

Decades after the demonstration of the direct effect of DNA methylation on gene expression, Howard Cedar continues to contribute to the field and is still active as professor emeritus. For instance, Cedar and his team have incorporated next-generation-sequencing based methods to interrogate DNA methylation dynamics involved in regulation of gene expression during development and disease (Falick Michaeli et al., 2024; Falick Michaeli et al., 2022; Radwan et al., 2024) and have looked at how genetic variation affects the epigenome in a variety of cell types in both mice and humans (Rosenski et al., 2025). Beyond his extensive and impactful work on DNA methylation in regulation of gene expression, Cedar has also made groundbreaking discoveries in other fields such as cancer biology, DNA replication as well as provide insights into the co-regulation of DNA methylation dynamics and asynchronous replication. His work often combines biological insight and development of innovative techniques to address impactful questions in a rigorous manner. In addition, Cedar has trained and mentored numerous productive scientists who now lead research teams, many of them continue to focus on epigenetics and DNA methylation and combine interdisciplinary approaches. Altogether, a wide range of studies and research avenues that have emerged by following and extending the path that was paved by the trailblazing work of Howard Cedar, continues to advance deeper understanding of the epigenetic and epigenomic regulation in health and disease.
